# Targeted Therapy in Nonmelanoma Skin Cancers

**DOI:** 10.3390/cancers3022255

**Published:** 2011-05-03

**Authors:** Giulia Spallone, Elisabetta Botti, Antonio Costanzo

**Affiliations:** Department of Dermatology, University of Rome “Tor Vergata”, Via Montpellier 1, 00199, Rome, Italy; E-Mails: giuliaspallone@hotmail.it (G.S.); elisabetta.botti@uniroma2.it (E.B.)

**Keywords:** basal cell carcinoma (BCC), squamous cell carcinoma (SCC), EGFR, SMO inhibitor, COX2

## Abstract

Nonmelanoma skin cancer (NMSC) is the most prevalent cancer in light-skinned populations, and includes mainly Basal Cell Carcinomas (BCC), representing around 75% of NMSC and Squamous Cell Carcinomas (SCC). The incidence of these tumors is continuously growing. It was found that the overall number of procedures for NMSC in US rose by 76%, from 1,158,298 in 1992 to 2,048,517 in 2006. Although mortality from NMSC tends to be very low, clearly the morbidity related to these skin cancers is very high. Treatment options for NMSC include both surgical and nonsurgical interventions. Surgery was considered the gold standard therapy, however, advancements in the knowledge of pathogenic mechanisms of NMSCs led to the identification of key targets for drug intervention and to the consequent development of several targeted therapies. These represent the future in treatment of these common forms of cancer ensuring a high cure rate, preservation of the maximal amount of normal surrounding tissue and optimal cosmetic outcome. Here, we will review recent advancements in NMSC targeted therapies focusing on BCC and SCC.

## 1. Introduction

In the USA, there are more new cases of skin cancer each year compared with the combined incidence of cancers of breast, prostate, lung, and colon cancers, with more than 1.5 million skin cancers diagnosed yearly in the United States. One in five Americans will develop skin cancer in the course of their lifetime. These and other epidemiologic findings strongly justify the development of novel, especially noninvasive therapeutic approaches for skin malignancies.

Skin cancer, including malignant melanoma, basal cell carcinoma (BCC), and squamous cell carcinoma (SCC), is the most common human cancer and represents a major public health concern due to its high incidence and the medical costs, mortality, and cosmetic associated deformities.

**Figure 1.**
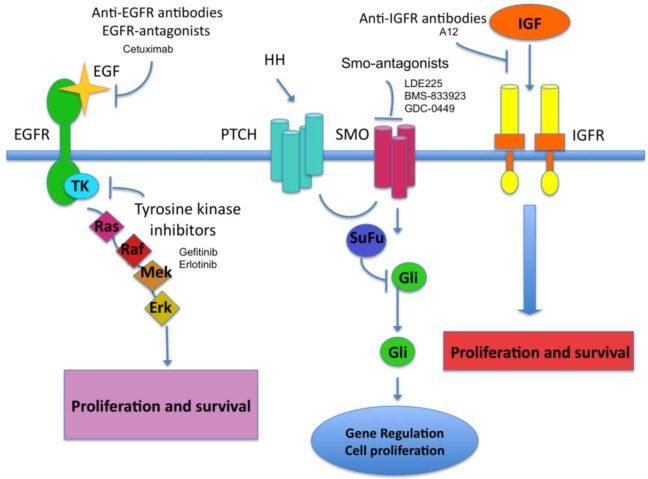
Schematic representation of molecular pathways targeted by drugs used in NMSC treatment.

In this paper we will review new therapeutic pharmacological approaches for nonmelanoma skin cancer (NMSC) that are based on molecular evidences accumulated in the past years (Figure 1). In particular, we will focus on inhibitors of critical molecular pathways involved in the development of BCC (Hedgehog-SMO/PATCHED-Gli) and cutaneous SCC (cSCC)(EGFR/ERBB2-MAPK).

## 2. Results and Discussion

### 2.1. COX-2 Inhibitors

Cyclooxygenase (COX), the rate-limiting enzyme for the production of prostaglandins (PG) from arachidonic acid, exists in at least two isoforms, COX-1 and COX-2. COX-1 is constitutively expressed while COX-2 expression is induced by inflammatory stimuli, such as ultraviolet light exposure. Increasing evidence is pointing to the role of COX-2 and its products, notably prostaglandin E2, in the development of NMSC. Overexpression of COX-2 has been revealed in various neoplasms ranging from colorectal cancer to breast cancer, as well as skin cancer [1,2].

Normal skin has minimal levels of COX-2 and PGE-2, with levels of COX-2 increasing in correlation with the severity of skin tumors from premalignant actinic keratosis to squamous cell carcinoma [2,3]. Studies have shown positive results with NSAIDs for the treatment of cancer by inhibiting angiogenesis and stimulating apoptosis mainly via COX-2 inhibition [3]. However, the nonspecific inhibition of COX-1 and COX-2 by NSAIDs leads to side effects associated predominantly with COX-1 inhibition, such as gastrointestinal ulcers and/or decreased renal function.

Selective inhibition of COX-2 is preferable to non-selective inhibition, because it selectively reduces cancer cell proliferation with minimal damage to the gastrointestinal tract. A specific cyclooxygenase-2 (COX-2) inhibitor, celecoxib, has shown potential therapeutic benefit in the prevention of cutaneous neoplasia.

Oral and topical celecoxib have demonstrated a chemopreventative effect in animal studies by inhibiting new tumor formation and delaying tumor latency [4,5]. These COX-2 specific inhibitors are promising agents in the battle against cutaneous neoplasia. Further double-blinded, randomized, placebo-controlled trials in humans are warranted to define the role of celecoxib and other COX-2 inhibitors in the prevention and treatment of NMSC, as well as their possible side effects. At the present moment a randomized phase II trial to determine the effectiveness of celecoxib in preventing Basal Cell Carcinoma in Patients with Basal Cell Nevus Syndrome has been completed and a trial on the use of Celecoxib in preventing Skin Cancer in patients with actinic keratosis is still ongoing.

### 2.2. Smoothened Homologue (SMO) Inhibitors

Basal-cell carcinoma is associated with mutations in components of the hedgehog signaling pathway [6-7]. Hedgehog (HH), a key regulator of cell growth and differentiation during development, controls epithelial and mesenchymal interactions in many tissues during embryogenesis. The HH signaling pathway is named after the family of extracellular HH ligands, of which there are three in mammals: sonic hedgehog (SHH), Indian hedgehog (IHH) and desert hedgehog (DHH). Extracellular hedgehog protein binds to patched homologue 1 (PTCH1), a 12-transmembrane receptor, and prevents PTCH1-mediated inhibition of signaling by smoothened homologue (SMO), a 7-transmembrane protein. PTCH1 is the receptor to which the HH ligands bind, and such binding relieves the inhibition of the pathway induced by unbound PTCH1, specifically through SMO in a non-stoichiometric manner. Once relieved of inhibition, SMO sends signals through a series of interacting proteins, including suppressor of fused (SUFU), culminating in activation of the downstream Gli family of transcription factors, GlI1, GlI2 and GlI3, the founding member of which was identified as a gene amplified in glioblastoma [6] with consequent induction of hedgehog target genes. Most basal-cell tumors have mutations in the hedgehog signaling pathway that inactivate PTCH1 [7-9] (loss-of-function mutation) or, less commonly, constitutively activate SMO15 (gain-of-function mutation) [6,7]. Nearly all sporadic BCC harbor mutations in components of the HH-induced pathway; PTCH1 and SMO being the most frequently mutated genes [10-12]. These mutations cause constitutive hedgehog pathway signaling, which in basal-cell carcinomas can mediate unrestrained proliferation of basal cells of the skin. For this reason, blocking the hedgehog pathway may be useful in treating patients with basal-cell carcinoma [14-16]. In general, BCCs seem to have relatively stable genomes—the few published studies suggest that they have lower levels of genomic instability than do many extracutaneous cancers [6,17]. As noted above, BCCs routinely carry mutations in PTCH1 and TP53 and, in 10% of instances, in SMO [18,19]. The mutations identified in PTCH1 and TP53 are frequently of a type that is consistent with their having been produced by UV radiation. This is true for BCCs that arise sporadically, and even more so for those large numbers of BCCs that arise in patients with xeroderma pigmentosum (XP), suggesting that repair of UV-induced DNA damage normally does reduce BCC carcinogenesis [20-22]. Furthermore, this suggests that one reason for the increased incidence of BCCs in older people might be the reported reduction of DNA repair with ageing [23]. However, there is one rare heritable disorder in which patients have a marked susceptibility to developing BCCs. This is basal-cell nevus syndrome (BCNS, also known as Gorlin syndrome or nevoid basal-cell carcinoma syndrome. Using family-based linkage studies of kindreds with BCNS, the locus carrying the causative mutant gene was mapped to human chromosome 9q22 [24] and then to the patched 1 (PTCH1) gene [9,25,26].

The novel SMO inhibitor GDC-0449 (2-chloro-N-(4-chloro-3-(pyridin-2-yl)phenyl)-4-(methylsulfonyl) benzamide) was discovered by high-throughput screening of a library of small-molecule compounds and subsequent optimization through medicinal chemistry GDC-0449 is a selective hedgehog pathway inhibitor. GDC- 0449 has antitumor activity in a mouse model of medulloblastoma and in xenograft models of primary human tumor cells, including colorectal cancer and pancreatic carcinoma, in which its effects correlate with blockade of the hedgehog pathway [27-29].

A phase 1 trial to evaluate the safety and adverse-effect profile of daily oral administration of GDC-0449 in patients with metastatic or locally advanced basal-cell carcinoma and other solid tumors was conducted by Von Hoff *et al.* Antitumor activity was observed in the first two patients with basal-cell carcinoma, prompting enrollment of additional patients to evaluate the activity and safety of the drug. Of the 33 patients, 18 had an objective response to GDC-0449, according to assessment on imaging (7 patients), physical examination (10 patients), or both (1 patient). Of the patients who had a response, 2 had a complete response and 16 had a partial response. There were no dose-limiting toxic effects or grade 5 adverse events, and only one grade 4 adverse event occurred during continuous daily administration of GDC-0449 for up to19 months. Those findings could confirm the participation of the hedgehog pathway in basal-cell carcinoma and suggest that inhibition of the hedgehog pathway can be useful in treating inoperable tumors.

Currently ongoing trials exist also for Smo—antagonists LDE225 selective smoothened antagonists, that bind to the Hedgehog (Hh)-ligand cell surface receptor. LDE225 is topically applied (either 0.25% or 0.75% LDE225) twice daily in an open-label manner for 6 or 9 weeks [30]. Smo-antagonist BMS-833923 is also object of study. As trials for both drugs are still ongoing, clinical results are still unclear [31].

### 2.3. Anti-EGFR Agents

cSCC encompasses 20% of nonmelanoma skin cancers, with an annual incidence of 200,000 to 300,000 in the United States [32]. Resection is typically curative, and the majority of cSCCs do not metastasize, but in the presence of certain risk factors, including location (e.g. Lip and ear), size (>2 cm in diameter), immunosuppression, association with scar or chronic wounds, and certain histopathologic features (depth of more than 4 mm, involvement of the reticular dermis or subcutaneous fat, or penetration into fascia, muscle, bone, or cartilage), metastatic rates range between 5% and 45% [33-35]. Few treatment options are available for recurrent or metastatic cSCC. Although cisplatin may be used as an initial treatment, it carries significant morbidity, including myelosuppression in 25% to 30%, dose-cumulative peripheral neuropathy in 30% to 100%, sensorineural hearing loss in 30%, dose-cumulative nephrotoxicity in 25% to 36%, and severe emesis in 100% of patients [36]. Furthermore, data supporting its efficacy are limited to small case series with no definitive long-term improvement in mortality [37-40]. A phase III trial also examined the use of retinoids in aggressive SCC,with no improvement in outcome [41]. With no established first-line agents for recurrent or metastatic cSCC, interest in the use of targeted therapies has grown. One promising target is EGFR, a transmembrane cell surface receptor with a tyrosine kinase domain whose overexpression promotes tumor survival and progression. Ligand binding activates EGFR, causing cell death inhibition, promotion of cell growth and proliferation, and angiogenesis. Cetuximab (Erbitux, Merck KGaA, Darmstadt, Germany) is a chimeric human-murine monoclonal antibody that competitively inhibits EGFR, boosting apoptosis and decreasing cellular proliferation, angiogenesis, and tumor invasion [42-45]. The 170-kDa EGFR is one of four members of the erbB family of transmembrane cell receptor tyrosine kinases. EGFR triggers downstream multilayered signaling pathways including the mitogen-activated protein kinase pathway, the phosphatidylinositol-3-kinase/Akt pathway and the Jak/Stat pathway [46,47]. These pathways, when abnormally activated in malignant cells, result in increased cancer cell proliferation, reduced apoptosis, and enhanced invasion and angiogenesis potentials [47,48]. EGFR is expressed in 15–30% of all breast cancers and in 20–40% of those with HER-2 overexpression [49,50]. EGFR expression is histologically defined as strong membranous staining in more than 10% of tumor cells.

Identification of the importance of the Erbb2 family member and signaling partner epidermal growth factor receptor (EGFR) on UV-induced skin tumors strongly supports a role for Erbb2 in skin tumorigenesis as well.

As mentioned above, the UV-induced activation of EGFR blocks cell cycle arrest, increases cell proliferation, suppresses apoptotic cell death, and increases skin tumorigenesis [49,50]. The effects of EGFR on apoptosis and cell cycle arrest result, at least in part, from its activation of phosphatidyl inositol-3-kinase (PI3K)/Akt signaling [47,49]. For example, EGFRdependent PI3K/Akt activation blocks the activation of signaling downstream from ataxia telangiectasia and Rad3-related (ATR) to block cell cycle arrest [47,51-53]. Activation of the ATR cell cycle checkpoint following UV-induced DNA damage allows time for DNA repair. ATR phosphorylates and activates Chk1, and to a lesser extent Chk2, kinases that phosphorylate the cell cycle regulator Cdc25a [54]. Phosphorylation by Chk1/2 inactivates Cdc25a and targets it for rapid, ubiquitin- directed degradation [55,56]. The Cdc25a phosphatase activates cyclin-dependent kinase (CDK)2 by removal of inhibitory phosphate groups at CDK2-Tyr15 and CDK2-Thr14 [55,56]. Loss of Cdc25a activity results in cell cycle arrest that allows time for the repair of DNA damage and reduces mutagenesis. If cell cycle arrest and DNA repair mechanisms are inadequate, cells acquire mutations that lead to cancer. EGFR promotes G2/M-phase progression by blocking the activation of this cell cycle checkpoint through PI3K/Akt-dependent inhibitory phosphorylation of Chk1 [57], a potential mechanism for its role in promoting UV-induced skin tumorigenesis [58]. In contrast to the extensive literature documenting the effects of EGFR activation on the response of the skin to UV, little investigation of the importance of other EGFR family members in UV-induced skin carcinogenesis has been undertaken. Madson *et al* hypothesized that repeated activation of the Erbb2 receptor resulting from chronic exposure to UV might also contribute to UV-induced skin tumorigenesis by deregulating cell cycle checkpoint control. This paradigm does not require oncogenic activation of Erbb2, but rather depends on repeated cycles of activation of normal physiological levels of proto-oncogenic Erbb2. Inhibition of the UV-induced activation of Erbb2 substantially reduced skin tumorigenesis in a transgenic mouse model [59]. Using both mouse skin and cell culture models, association of UV-induced skin tumorigenesis with Erbb2-dependent inhibitory phosphorylation of Chk1, maintenance of Cdc25a, and decreased cell cycle arrest was documented. These data could demonstrate that activation of Erbb2 on UV irradiation increases UV-induced skin tumorigenesis by suppressing a DNA damage-induced cell cycle checkpoint.

Anti-EGFR agent cetuximab is currently approved for the treatment of irinotecan-refractory metastatic colorectal cancer and for that of head and neck squamous-cell carcinoma in association with radiotherapy [46,60,61]. Cetuximab compete with TGF-α, EGF and other natural ligands for EGFR ectodomain thus preventing autophosphorylation of the intracellular region, inhibiting ligand-dependent activation of the EGFR, resulting in an aborted dimerization with other erbB receptors, and subsequently, EGFR internalization and inhibition of downstream signaling pathways [47]. As a result, the TK domain remains inactive and downstream signaling does not occur, which leads to inhibition of cell cycle progression, promotion of apoptosis, and antiangiogenesis.

Cetuximab can also elicit antitumor activity by antibody-dependant cell cytotoxicity [51]. Paclitaxel prevents cell replication by stabilizing microtubule bundles during mitosis [47]. The most likely hypothesis for the enhanced antitumor activity of combined cetuximab+paclitaxel is an increase in cancer cell apoptosis coupled with a decrease in cell proliferation. Studies on cancer cell cultures and in human tumor xenografts showed that paclitaxel upregulates EGFR and HER-2 receptors, and renders cancer cells more susceptible to cetuximab and trastuzumab, respectively [53,61]. Cetuximab is approved for the treatment of locally or regionally advanced head and neck cancer, of metastatic or recurrent squamous cell carcinoma, refractory to platinum-based therapy, as monotherapy or in combination with radiation therapy; it is also approved for the treatment of metastatic colorectal cancer CC [64], EGFR-expressing, as monotherapy in patients refractory to irinotecan-based chemotherapy, or in combination with Irinotecan and in the treatment of non small cell lung cancer (NCSLC).

Cetuximab has been shown to circumvent tumor resistance to chemotherapy agent irinotecan in some colorectal cancer patients [60]. Mechanisms such as drug efflux abrogation, apoptosis restoration and impairment of DNA-repair activity in cancer cells have been proposed to explain this phenomenon [62,63]. Clinical trials using cetuximab as a first-line or second-line therapy for patients with cSCC with in-transit or metastatic disease may help to define its potential role in managing this difficult population.

Adding cetuximab to radiotherapy also improves disease control and overall survival in locoregionally advanced lSCCHN. [61,65]. Similar improvements in overall survival of patients with recurrent or metastatic SCCHN were noted by combining cetuximab with standard platinum-based regimens and by using it as a single second line agent in patients who failed standard therapy [65-68]. Statistically significantly greater overall survival has also been reported after adding cetuximab to the standard platinum-based chemotherapy used to treat NSCLC and patients with a squamous cell histologic subtypewere found to retain survival benefit in subgroup analysis [69].

A pruritic erythematous papulopustular eruption can develop within 2 to 3 days of starting cetuximab treatment, which may be associated histologically with a neutrophil-predominant upper dermal infiltrate [68,69]. This eruption is the most common side effect, which occurs in 76% to 88% of patients, is rarely dose limiting, and typically resolves with discontinuation of treatment [33,43,65]. Rash severity is possibly predictive of a favorable clinical outcome, because prospective trials in SCCHN and CC have shown a survival advantage in patients who developed cetuximab-related skin toxicity over those who did not [66,72]. EGFR is known to be expressed in cSCC, although it is likely that the pattern of carcinogenesis is complex. It is unclear whether expression level correlates with prognosis or tumor response to targeted therapy. Several studies show that EGFR is expressed more in locoregional and distant metastases than in primary sites and suggest that upregulation promotes tumor aggression [73-75]. In contrast, a retrospective analysis found no evidence that EGFR expression level is an independent prognostic indicator [76]. Despite this, EGFR-targeted therapy is being validated as an effective approach to therapy in cSCC.

Additional case reports show promising responses to cetuximab in patients with metastatic cSCC. One report noted a complete clinical response to weekly infusions of single-agent cetuximab in a patient with multiple nodal metastases from a poorly differentiated primary cSCC who had failed several cycles of palliative radiation therapy. His disease recurred upon discontinuation of cetuximab, but he again achieved a complete clinical response when weekly infusions were restarted. A second report described complete and near-complete clinical responses to weekly infusions of cetuximab in two patients with extensive cSCC recurrence and intransit metastases not responsive to radiation treatment [77,78]. Additionally, two recent case reports noted partial and complete clinical response in patients with metastatic cSCC who had previously failed multiple platinum-based chemotherapy regimens and radiation treatment. One described partial clinical response with 50% reduction in size of a metastatic node, which was maintained for 11 months on weekly cetuximab infusions before disease progression occurred. A second reported 3-month progression free survival on weekly cetuximab infusions, which are still ongoing. Miller *et al.* reported a case of complete clinical response using cetuximab as first-line treatment for cSCC with in-transit and distant nodal metastases [43]. In this case Cetuximab was preferred to cisplatin and considered a suitable alternative because of its relative lack of side effects and ease of administration.

Most common adverse effects of the EGFR-blockade are skin toxicities, that with Cetuximab can reach over 80% of frequency, including acne-like rush, xerosis cutis, paronychia and fissuring, hair changes and mucositis. Cetuximab‘s side-effect profile includes hypersensitivity reactions, nausea, vomiting, diarrhea, abdominal pain, and rash. Approximately 2% to 5% of patients experience grade 3 to 4 infusion reactions, usually after the first dose, although this has been fatal in less than 1 in 1,000 cases and can be ameliorated by premedication with corticosteroids and diphenhydramine. In addition, 30% of patients experience fever, 80% fatigue, 59% abdominal pain, 37% vomiting, and 39% diarrhea. Rare adverse events include case reports of acute cytotoxic dermatitis after receiving cetuximab in the setting of concurrent radiation therapy, suggesting possible overlap between radiation dermatitis and cetuximab-induced rash [79,80]. Fatal diffuse alveolar damage has also been reported in two lung transplant patients receiving cetuximab for metastatic cSCC. Although approximately 1% of patients receiving the EGFR inhibitors gefitinib and erlotinib develop interstitial lung disease, no studies have linked this side effect to cetuximab. Because there are no data in the literature concerning use of EGFR inhibitors in people who have received solid organ transplants, these agents should probably be used with caution in this subset of patients.

Furthermore, other anti-EGFR antibodies have shown efficacy against cSCC. EGFR antagonist Erlotinib and Gefitinib are tyrosine kinase inhibitor FDA approved on November 2004 for Lung cancer – Non Small Cell (Accelerated Approval Program AAP). The inhibitors of TK phosphorylation (TK inhibitors [TKIs]) are small-molecule agents that block EGFR activity by interfering with the adenosine triphosphate-binding site on the intracellular region of the receptor. Erlotinib is indicated in monotherapy for the treatment of patients with locally advanced or metastatic NSCLC after failure of at least one prior chemotherapy regimen. Apparently, no survival benefit or other clinically relevant effects have been demonstrated in patients with EGFR- negative tumours. Erlotinib in combination with gemcitabine is indicated for the treatment of patients with metastatic pancreatic cancer. The tyrosine kinase inhibitor Gefitinib was approved on May, 2003 for locally advanced or metastatic nonsmall Lung cancer-NSCLC (AAP) with activated mutations of EGFRTK. Gefitinib has been shown to induce radiographic tumor responses, improve symptoms, and improve quality of life in those patients who have failed to respond to previous cancer therapies [45]. Gefitinib has produced a 15% partial response rate and 45% stable disease rate in a prospective trial of patients with recurrent or metastatic SCC [37,45]

Recent success involving the therapeutic use of antibodies and small molecule inhibitors against tyrosine kinases have generated considerable interest in research aimed at targeting these receptors in a wide variety of malignancies. In an attempt to improve the treatment of cSCC, Galer [81] explored the effect of inhibition of two of these receptors on cutaneous tumor growth *in vitro* and *in vivo*. The insulin-like growth factor-I receptor(IGF-IR) is a ubiquitous transmembrane tyrosine kinase composed of two extracellular alpha subunits and two intracellular beta subunits [82-83]. Ligand binding (IGF1 or IGF2) to the extracellular alpha subunits, trigger conformational changes in the beta subunits activating the receptors tyrosine kinase activity, which in turn activates downstream signaling cascades, including the phosphatidylinositol 3-kinase/AKT and Ras/Raf/mitogen-activated protein kinase (MAPK) pathways [84-90]. Numerous human tumors have been shown to overexpress IGFIR or have increased IGF-IR kinase activity. Targeted therapies, including insulin-like growth factor (IGF) binding proteins, human monoclonal antibodies, and small-molecule tyrosine kinase inhibitors against IGF-IR, have been developed and show promise for therapeutic use in both *in vitro* and *in vivo* experiments [91-93]. A12, a high-affinity human monoclonal antibody to IGF-IR, has been shown to induce apoptosis and inhibit tumor growth by competitively binding to the receptor and inducing IGF-IR internalization and downregulation. Experimentally, A12 has been shown to inhibit the growth of breast, pancreatic, colon, and renal tumors, both *in vitro* and *in vivo* with little toxicity or weight loss in nude mouse models [94]. Galer *et al* [81] hypothesize that targeted therapy against IGF-IR (A12) and EGFR (cetuximab) will inhibit CSCC tumor growth *in vitro* and in an athymic nude mouse model. Our findings indicate that the combination ofA12 and cetuximab simultaneously blocks EGFR and IGF-IR activation and significantly reduces tumor volume by both direct antitumor and angiogenic effects. We showed that elevated IGF-IR and EGFR expression is consistently and concurrently elevated in CSCC cell lines. In an orthotopic nude mouse model of CSCC, dual inhibition with A12 and cetuximab reduced the tumor volume by 92% as compared to approximately 50% with either agent alone. Combination treatment also significantly improved survival. *In vitro* studies demonstrated the inhibition of activation of IGF-IR by A12 and EGFR by cetuximab, but showed no cross-inhibition. The combination of A12 and cetuximab also showed direct growth inhibitory and apoptotic activity against CSCC cell lines. *In vivo* monotherapy with either A12 or cetuximab caused a significant increase in apoptosis and a decrease in both cellular proliferation and microvessel density as compared to the control group, an effect which was enhanced for combination treatment. A12 treatment resulted in downregulation of the total IGF-IR expression levels, as has been demonstrated by others [95]. Treatment with A12 and cetuximab significantly inhibited SCC tumor growth in the murine model. Either agent alone resulted in approximately a 50% reduction in tumor volume, whereas treatment with a combination of the two drugs resulted in a greater than 90% reduction in tumor volume. Similarly, longer survival was also found for the combined treatment group as compared with the control or A12 treatment groups. However, the concurrent expression of both IGF-IR and EGFR together in CSCC seems to be important for tumor growth and development, and simultaneous inhibition of these two tyrosine kinases results in a significantly greater reduction of tumor development and growth. Our findings are consistent with other studies suggesting that targeted therapy of IGF-IR can be used in combination with other therapeutic strategies to achieve maximum antitumor effects [96,97] and that combination therapy may be beneficial in preventing the development of drug resistance, such as that seen with trastuzumab (Herceptin), erlotinib, and gefitinib [97-99]. Immunohistochemical analyses of tumor sections from mice treated with either A12 or cetuximab alone revealed a significant decrease in the proliferative index as measured by PCNA staining and an increase in intratumoral apoptosis as measured by the TUNEL assay. This effect was strongest for groups treated withA12, either alone or in combination, which is in agreement with previous work showing only moderate apoptosis in response to cetuximab treatment alone but synergism with dual agent therapy. The increase in intratumoral apoptosis and decrease in proliferation is in contrast to our *in vitro* findings showing limited effects of either agent or the combination on the growth of Colo16 cells. The differences between the results from the animal model and *in vitro* experiments can be explained by reports showing the activation of vascular endothelial growth factor receptor (VEGFR) signaling by IGFR in cancer [100]. Recent studies have demonstrated that both EGFR and IGF-IR are abundantly expressed on endothelial cells [83,101]. Insulin-like growth factor I receptors are more abundant than insulin receptors in human micro- and macrovascular endothelial cells. [102]. Vascular endothelial growth factor C expression and lymph node metastasis are regulated by the type I insulin-like growth factor receptor [103,104].

EGF and IGF produced at high levels by tumors are able to promote the growth, survival, and migration of tumor cells, and induce the synthesis of VEGF-A, VEGF-C, and MMP2, which may enhance the development of the blood supply essential for the progressive growth of primary malignancies and their metastases [105-107].Treatment with eitherA12 or cetuximab alone resulted in statistically significant inhibition of tumor-associated angiogenesis, whereas the combination treatment with A12 and cetuximab resulted in an additional inhibition of angiogenesis. These findings concur with other studies that have demonstrated reduction of angiogenesis resulting from inhibition of IGF-IR, EGFR, or both receptors simultaneously [83,107]. Current studies have indicated that the efficacy of tyrosine kinase inhibition can be enhanced by combining it with other tyrosine kinase inhibitors, chemotherapy, or radiotherapy [107,109-112]. Although a demonstrable response was achieved using A12 or cetuximab as single agents, the enhanced response obtained when these two monoclonal antibodies were used in combination provides further support for the use of not only multiple tyrosine kinase inhibitors, but the use of inhibitors of IGF-IR and EGFR in particular [113-116]. This agrees with previous works showing a synergistic effect of the combination of IGF-IR and EGFR inhibition and suggests the presence of “cross-talk” between the receptors [117].

In summary, dual inhibition of the tyrosine kinases EGFR and IGF-IR can decrease skin cancer growth both *in vitro* and *in vivo*. These data suggest that dual inhibition of tyrosine kinases, EGFR and IGF-IR in particular, may be therapeutically useful and provide a promising strategy for the treatment of patients with aggressive CSCC [80].

## 3. Conclusions

NMSC continues to increase in prevalence. Treatment options employed should be tailored to the type of tumor, tumor location, size of tumor, and histologic pattern. Surgical methods remain the ‘gold standard’ although alternatives to surgery are appropriate in certain tumors if considerable disfigurement or functional impairment might result from surgery. In addition to longstanding nonsurgical options such as radiation therapy and cryosurgery, the newer additions of biologic immune response modifiers (e.g., imiquimod) and photodynamic therapy can be used in select tumors and can reduce morbidity. Emerging treatments include several drugs belonging to the so-called targeted therapies. In particular, Hedgehog pathway inhibitors for BCC, EGF pathway inhibitors for AK and SCC, as well as Cox2 inhibitors for AK, represent new stimulating options for clinical management of these common malignancies.
